# Synthesis, secretion, function, metabolism and application of natriuretic peptides in heart failure

**DOI:** 10.1186/s13036-017-0093-0

**Published:** 2018-01-12

**Authors:** Shihui Fu, Ping Ping, Fengqi Wang, Leiming Luo

**Affiliations:** 1 Department of Geriatric Cardiology, Chinese People’s Liberation Army General Hospital, Beijing, 100853 China; 2 Department of Cardiology and Hainan Branch, Chinese People’s Liberation Army, General Hospital, Beijing, China; 3 Department of Pharmaceutical Care, Chinese People’s, Liberation Army General Hospital, Beijing, China

**Keywords:** Cardiac precursor cells, Dipeptidyl peptidase-4, Heart failure, Insulin degrading enzyme, Angiotensin receptor blocker neutral endopeptidase inhibitor, micro-RNA, Natriuretic peptides, Nesiritide, Designer natriuretic peptides, Natriuretic peptide precursor

## Abstract

As a family of hormones with pleiotropic effects, natriuretic peptide (NP) system includes atrial NP (ANP), B-type NP (BNP), C-type NP (CNP), dendroaspis NP and urodilatin, with NP receptor-A (guanylate cyclase-A), NP receptor-B (guanylate cyclase-B) and NP receptor-C (clearance receptor). These peptides are genetically distinct, but structurally and functionally related for regulating circulatory homeostasis in vertebrates. In humans, ANP and BNP are encoded by NP precursor A (NPPA) and NPPB genes on chromosome 1, whereas CNP is encoded by NPPC on chromosome 2. NPs are synthesized and secreted through certain mechanisms by cardiomyocytes, fibroblasts, endotheliocytes, immune cells (neutrophils, T-cells and macrophages) and immature cells (embryonic stem cells, muscle satellite cells and cardiac precursor cells). They are mainly produced by cardiovascular, brain and renal tissues in response to wall stretch and other causes. NPs provide natriuresis, diuresis, vasodilation, antiproliferation, antihypertrophy, antifibrosis and other cardiometabolic protection. NPs represent body’s own antihypertensive system, and provide compensatory protection to counterbalance vasoconstrictor-mitogenic-sodium retaining hormones, released by renin-angiotensin-aldosterone system (RAAS) and sympathetic nervous system (SNS). NPs play central roles in regulation of heart failure (HF), and are inactivated through not only NP receptor-C, but also neutral endopeptidase (NEP), dipeptidyl peptidase-4 and insulin degrading enzyme. Both BNP and N-terminal proBNP are useful biomarkers to not only make the diagnosis and assess the severity of HF, but also guide the therapy and predict the prognosis in patients with HF. Current NP-augmenting strategies include the synthesis of NPs or agonists to increase NP bioactivity and inhibition of NEP to reduce NP breakdown. Nesiritide has been established as an available therapy, and angiotensin receptor blocker NEP inhibitor (ARNI, LCZ696) has obtained extremely encouraging results with decreased morbidity and mortality. Novel pharmacological approaches based on NPs may promote a therapeutic shift from suppressing the RAAS and SNS to re-balancing neuroendocrine dysregulation in patients with HF. The current review discussed the synthesis, secretion, function and metabolism of NPs, and their diagnostic, therapeutic and prognostic values in HF.

## Background

As a family of hormones with pleiotropic effects, natriuretic peptide (NP) system includes atrial NP (ANP), B-type NP (BNP, also called brain NP), C-type NP (CNP), dendroaspis NP (DNP) and urodilatin, with three receptors: NP receptor-A [guanylate cyclase (GC)-A or NPR-A], NP receptor-B (GC-B or NPR-B) and NP receptor-C (clearance receptor or NPR-C) [1]. These peptides are genetically distinct, but structurally and functionally related for regulating circulatory homeostasis in vertebrates, and each of them has a 17-amino acid (aa) cyclic structure constructed with an disulfide bond [2]. In humans, ANP and BNP are encoded by NP precursor A (NPPA) and NPPB genes on chromosome 1, whereas CNP is encoded by NPPC on chromosome 2 [3]. NPs are synthesized and secreted through certain mechanisms by cardiomyocytes, fibroblasts, endotheliocytes, immune cells (neutrophils, T-cells and macrophages) and immature cells, such as embryonic stem cells, muscle satellite cells and cardiac precursor cells (CPCs) [4]. They are mainly produced by cardiovascular, brain and renal tissues in response to wall stretch and other causes. NPs provide natriuresis, diuresis, vasodilation, antiproliferation, antihypertrophy, antifibrosis and other cardiometabolic protection [5, 6]. More importantly, NPs represent body’s own antihypertensive system, and provide compensatory protection to counterbalance vasoconstrictor-mitogenic-sodium retaining hormones, released by renin-angiotensin-aldosterone system (RAAS) and sympathetic nervous system (SNS) [7]. NPs are inactivated through not only NPR-C, but also neutral endopeptidase (NEP), dipeptidyl peptidase-4 (DPP-4) and insulin degrading enzyme (IDE). There is urinary excretion of NPs as well [3]. The current review discussed the synthesis, secretion, function and metabolism of NPs, and their diagnostic, therapeutic and prognostic values in heart failure (HF).

## Synthesis and secretion

### Synthesis and secretion of ANP

**Fig. 1:**
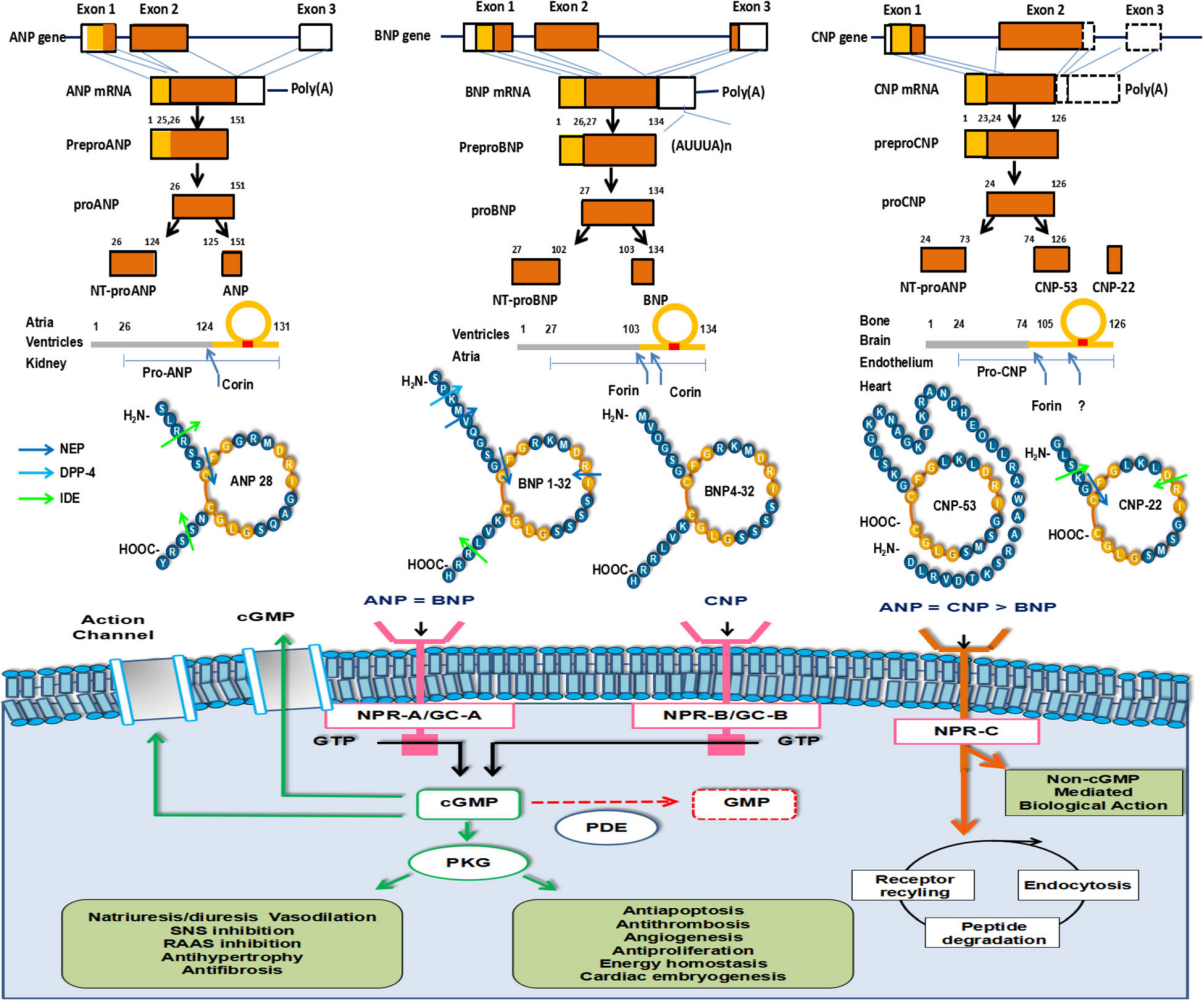
Synthesis, metabolism and function of natriuretic peptides. Abbreviations: ANP: atrial natriuretic peptide; BNP: B-type natriuretic peptide; cGMP: cyclic guanosine monophosphate; CNP: C-type natriuretic peptide; DPP-4: dipeptidyl peptidase-4; GC: guanylate cyclase; GTP: guanosine triphosphate; IDE: insulin degrading enzyme; NEP: neutral endopeptidase; NPR: natriuretic peptide receptor; NT-proANP: N-terminal proANP; NT-proBNP: N-terminal proBNP; NT-proCNP: N-terminal proCNP; PDE: phosphodiesterase; PKG: protein kinase; RAAS: renin-angiotensin-aldosterone system; SNS: sympathetic nervous system

ANP is mainly produced and stored in atrial granule, and normal ventricle actually produces little ANP [8]. Failing ventricle secretes ANP in patients with HF, and becomes a main part of plasma ANP [9]. NPPA gene has the following exons: exon 1 [5’-untranslated region (5’-UTR, a 25-aa signal peptide) and 16 aa of proANP sequence], exon 2 (most of proANP sequence) and exon 3 [terminal tyrosine and 3’-untranslated region (3’-UTR)] (Fig. 1). Proximal 5’-flanking region (5’-FR) of NPPA gene can regulate its spatio-temporal expression [10]. Mechanical stretch of cardiomyocytes, exercise, hypoxia, cold, angiotensin, endothelin, vasopressin, catecholamine or glucocorticoid induces transcription factor GATA to bind promoters, suggesting an active involvement of neurohormonal system in the regulation of NP synthesis. ANP mRNA is translated to 151-aa preproANP, and 126-aa proANP is produced and stored after removing 25-aa signal peptide. ProANP is cleaved upon secretion by transmembrane serine endoprotease (corin) into the active carboxy-terminal (C-terminal) 28-aa α-ANP with relatively short half-life, which can bind to receptor and have biologic function, and the inactive 98-aa amino-terminal (N-terminal) proANP (NT-proANP) more stable with relatively long half-life [11]. ANP secretion is caused by atrial and ventricular wall stretch due to transmural pressure or volume overload, and also affected by age, sex, heart rate and renal function [2]. ANP is distributed into coronary sinus, and then to various target organs. Meanwhile, human β-ANP, an antiparallel dimer of α-ANP, is present in failing heart, and has elevated levels in patients with severe HF [12]. Additionally, urodilatin is a 32-aa NP of renal origin with local function in regulating renal sodium and water excretion through interacting with NPR-A. It is produced as N-terminal 4-aa-extended form of α-ANP like γ-ANP after the cleavage of proANP by an unknown protease in renal distal tubules [13].

### Synthesis and secretion of BNP

Firstly discovered in porcine brain, BNP is stored in atrial granule with ANP, but not in granule in ventricle [14]. BNP is mainly secreted by normal atrium [15]. However, as a hallmark for maladaptive remodeling of left ventricle (LV), BNP is mainly secreted in ventricle when LV function is insufficient and cardiac wall is stretched due to transmural pressure or volume overload [16]. BNP has more obviously elevated levels than ANP in patients with HF [17]. NPPB gene has the following exons: exon 1 (5’-UTR, a 26-aa signal peptide, and 15 aa of proBNP sequence), exon 2 (most of proBNP sequence) and exon 3 (terminal tyrosine and 3’-UTR) (Fig. 1). Various causes, such as tissue hypoxia, transmural pressure or volume overload, induce the transcription of NPPB gene in endoplasmic reticulum to produce 134-aa preproBNP [18]. Pro-inflammatory cell factors, including interleukin-1β, interleukin-6 and tumor necrosis factor-α, induce BNP synthesis in cardiomyocytes, suggesting an active involvement of immune system in the regulation of NP synthesis [19]. Repeated AUUUA in 3’-UTR of BNP mRNA make it easier to be degraded than ANP mRNA. After removing 26-aa signal peptide, 108-aa proBNP is produced and then cleaved upon secretion by furin (or corin) into the active BNP1-32 (or BNP4-32), and the inactive 76-aa N-terminal proBNP (NT-proBNP) [20].

### BNP-32 and proBNP-108

Not only proBNP and O-Glycosylated proBNP, but also BNP and NT-proBNP, are present in blood [14]. Current immunoassays for BNP-32 also recognize proBNP-108, and plasma BNP in HF rather includes proBNP-108 and O-Glycosylated proBNP-108 [21]. There is an elevated ProBNP-108/BNP-32 ratio in patients with HF [22]. ProBNP-108/BNP-32 ratio increases in response to ventricular overload rather than atrial overload in patients with HF [23]. Moreover, proBNP-108 has much less ability to induce the synthesis of cyclic guanosine monophosphate (cGMP) than BNP-32 in vascular smooth muscle and endothelial cells [24]. Despite high levels of plasma BNP, there is an attenuation of BNP bioactivity in patients with HF [25]. In mild to moderate HF, plasma cGMP levels increase in proportion to HF severity. However, plasma cGMP levels have an attenuated increase relative to disease state, and no longer correlate with BNP levels in severe HF [26]. Even relatively small increase in proBNP-108/BNP-32 ratio, if it lasts for a long time, can sufficiently reduce total potential of cGMP production and attenuate compensatory benefit of plasma BNP, thereby leading to HF progression.

### O-Glycosylation and proBNP-108

O-Glycosylated proBNP-108 levels correlate with HF severity [24]. Within Golgi apparatus of ventricular myocytes, proBNP-108 is post-translationally glycosylated to various extent at seven sites of N-terminal region: threonine (Thr)36, serine (Ser)37, Ser44, Thr48, Ser53, Thr58 and Thr71 [27]. Within trans-Golgi network, O-Glycosylated proBNP-108 is cleaved into BNP-32 and NT-proBNP [23]. O-Glycosylation has potential effect on the secretion and processing of proBNP-108. Thr71 is near to cleavage site of proBNP-108, and O-Glycosylation at Thr71 inhibits processing of proBNP-108 in human embryonic kidney (HEK) 293 cells and human leukemia 1 cells [28, 29]. Mechanisms governing cardiac secretion and peripheral metabolism of proBNP-108 remain unclear in patients with severe HF [30]. Venous furin-like enzyme activity has been proposed to correlate with NTproBNP/proBNP-108 ratio, and cleave proBNP-108 into BNP-32 and NT-proBNP in peripheral blood of patients with acute HF [31]. However, it has also been reported that proBNP-108 has similar levels between peripheral vein and artery, and is unlikely to be metabolized in peripheral blood and tissue. These inconsistent results may be caused by the diversity between in vivo and in vitro settings, between glycosylated and non-glycosylated proBNP-108, and between clinical status of patients with HF [32].

### New forms of BNP

Many new plasma forms of BNP with low molecular mass are present, but BNP-32 is nearly absent in patients with HF [33]. DPP-4 cleaves N-terminal serine-proline dipeptide from BNP1-32 in blood to produce BNP3-32 and BNP5-32, which are more rapidly degraded and have much less abilities to induce beneficial responses than BNP1-32 [34]. DPP-4 also cleaves N-terminal histidine-proline dipeptide from proBNP1-108 to produce proBNP3-108. Current immunoassays can not distinguish these forms in blood [35]. Patients using DPP-4 inhibitors have exhibited an increased risk of HF hospitalization in Saxagliptin Assessment of Vascular Outcomes Recorded in patients with diabetes mellitus-Thrombolysis in Myocardial Infarction (SAVOR-TIMI) trial [36]. However, other studies have not confirmed an association between DPP-4 inhibitors and HF hospitalization and shown adverse prognosis in patients with HF using DPP-4 inhibitors [37–41]. Previous meta-analyses can not rule out the concern that DPP-4 inhibitors have cardioprotective roles by affecting BNP1-32/BNP3-32 ratio [42–45].

### Synthesis and secretion of CNP

As the most abundant NP in brain, CNP is also synthesized in atrium, ventricle, kidney, chondrocyte, endothelium and blood cells. NPPC gene has the following exons: exon 1 (5’-UTR, a 23-aa signal peptide, and 7 aa of proCNP sequence), exon 2 (most of proCNP sequence) and exon 3 (3’-UTR) (Fig. 1). After removing 23-aa signal peptide from 126-aa preproCNP, 103-aa proCNP is produced and then cleaved upon secretion into 53-aa CNP by intracellular serine endoprotease (furin) [46]. CNP expression is up-regulated by shear stress [47]. Transforming growth factor-β stimulates CNP production and secretion in endotheliocytes [2]. CNP-53 is then cleaved into 22-aa CNP, and CNP-53 and CNP-22 are equipotent [48]. Although CNP-22 and CNP-53 have similar activity and function, CNP-53 predominates in heart, endothelium and brain, whereas CNP-22 predominates in cerebral spinal fluid and plasma [49].

## Function

### Receptors of NPs

NP system has significant autocrine, paracrine and endocrine function. As two of five transmembrane GC receptors in humans, NPR-A and NPR-B induce pathophysiologic functions of NP system (Fig. 1). NPR-A is activated by ANP and BNP, and NPR-B is activated by CNP [50]. NPRs are present in heart, brain, kidney, adrenal, liver, pancreas, vascular and gastrointestinal smooth muscle, adipocytes, chondrocytes, fibroblasts and platelets [3]. NPR-A is highly present in kidney, adrenal, lung, terminal ileum, aorta and adipose. NPR-B is highly present in fibroblasts [51]. They have an extracellular ligand-binding and membrane-spanning region, an intracellular particulate GC region and an intracellular cGMP-dependent protein kinase (PKG) region [52]. Activated NPRs catalyze conversion of guanosine triphosphate (GTP) to cGMP. As an intracellular second messenger, cGMP activates PKG and phosphodiesterase (PDE) to regulate various pathways including ion channels, protein phosphorylation, nuclear translocation and gene expression, all of which exert biologic effects [53, 54]. NPR-C is highly present in atrium, kidney, adrenal, lung, mesentery, placenta, cerebral cortex, cerebellum, aorta and vein [51]. NPR-C has a disulfide-bonding dimer homologous to extracellular region of NPR-A and NPR-B and intracellular 37 aa for potential signaling functions. NPR-C is a clearance receptor for ANP, BNP and CNP [55]. Although NPR-C has no GC activity, it induces pathophysiologic functions, such as affecting cell growth, through adenylate cyclase, inhibitory guanine nucleotide-regulatory protein (G-protein) and phospholipase C [56]. Modulation of NPR expression in target organ may be determinant for local bioavailability and regulation of NPs. Therefore, NP resistance may be caused by NPR-C upnregulation or NPR-A downregulation. In early stage of HF, NPs provide compensatory actions including not only natriuresis, diuresis and vasodilation, but also RAAS and SNS inhibition. In severe HF, NPs have attenuated effects despite high plasma NP levels assessed by current immunoassays. Several possible explanations include increased NP degradation, reduced NP bioactivity, increased secretion of inactive NP forms, increased cGMP degradation and reduced NPR-A activity due to receptor dephosphorylation and degradation [57–59]. However, limited information is available regarding bioactivity change in NPR and its clinical significance in HF.

### Function of ANP

ANP boosts natriuresis and diuresis. Na^+^ reabsorption in inner medullary collecting ducts depends mainly on the apical amiloride-sensitive Na^+^ channel (cyclic nucleotidegated ion channel) and basolateral Na^+^-K^+^-adenosine triphosphatase (Na^+^-K^+^-ATPase). Apical amiloride-sensitive Na^+^ channel allows passive Na^+^ entry from renal tubular lumen. Basolateral Na^+^-K^+^-adenosine triphosphatase helps Na^+^-K^+^-2Cl^-^ cotransporter actively pump Na^+^ out of epithelial cell into peritubular space and eventually bloodstream. ANP inhibits apical Na^+^ channel and basolateral Na^+^-K^+^-ATPase activity, resulting in decreased Na^+^ reabsorption from inner medullary collecting ducts and increased Na^+^ excretion from urine known as natriuresis [53]. ANP induces cGMP to inhibit Na^+^ channel by a mechanism of activating PKG independent of phosphorylation [2]. ANP inhibits basolateral Na^+^-K^+^-ATPase through PKG-induced phosphorylation in a cGMP-dependent manner [53]. ANP inhibits RAAS with reduced angiotensin II-induced sodium and water transport in renal proximal tubules [60]. ANP suppresses renin secretion from juxtaglomerular (granular) cells in a cGMP-dependent manner without changing intracellular Ca^2+^. ANP also suppresses aldosterone synthesis in adrenal glomerulosa (adrenocorticotropic hormone-induced, angiotensin-II-induced and basic aldosterone), which enhances natriuretic effect and reduces extracellular volume [61]. ANP/NPR-A activity induces natriuresis and diuresis, but appears to be downregulated in HF with RAAS activation [62]. Although conflicting studies exist, ANP may suppress angiotensin-II-induced secretion of vasopressin from posterior pituitary without blood-brain barrier, and inhibit V2 receptor-mediated effect of vasopressin on water reabsorption in collecting ducts [63–65]. ANP increases glomerular filtration rate (GFR) through its direct vasodilative effect on afferent arterioles. ANP also reverses norepinephrine-induced vasoconstriction of afferent arterioles [66]. Vasodilative effect of ANP on vascular smooth muscle may involve following mechanisms: reducing Ca^2+^ influx, enhancing Ca^2+^ extrusion, and inhibiting Ca^2+^ release from sarcoplasmic reticulum [67]. ANP directly relaxes contractile intraglomerular mesangial cells and expands glomerular capillary surface area available for filtration. ANP also inhibits angiotensin-II-induced constriction of mesangial cells [67]. There have been controversies on vasoconstrictive effect of ANP on efferent arterioles: some have observed no change in diameter of efferent arterioles, whereas others have reported the dilatation of afferent arterioles and constriction of efferent arterioles [68].

### Lowering blood pressure

ANP induces hypovolemia and decreases blood pressure (BP). ANP lowers BP with increased permeability of capillaries and fluid efflux from blood [69]. ANP decreases cardiac load by shifting intravascular fluid into interstitial space. ANP stimulates Ca^2+^/calmodulin-dependent endothelial nitric oxide (NO) synthase in aorta, ventricle and kidney to produce more NO for relaxing vascular smooth muscle cells by binding to either NPR-A or NPR-C, causing a decrease in BP levels [70]. ANP decreases vascular resistance also by inhibiting RAAS [71]. Thus, ANP/NPR-A activity reduces basic BP levels through its combined effects on vascular relaxation and intravascular volume. Moreover, ANP relaxes air passages and blood vessels in lung [72]. ANP and BNP have elevated levels due to wall stretch of right ventricle, and inhibit pulmonary hypertension caused by chronic hypoxia [73].

### Counteracting sympathetic activity

ANP not only modulates baroreflex mechanism, but also inhibits sympathetic activity and enhances vagal afferent. SNS is inhibited in peripheral vessels, perhaps by lowering activation threshold of baroreceptors, by decreasing catecholamine release from nerve endings, and by reducing sympathetic outflow [74]. It lowers activation threshold of vagal afferent, reflex tachycardia and vascular constriction, and causes a sustained decrease in mean arterial pressure [75]. ANP decreases sympathetic outflow by modulating ganglionic neurotransmission rather than increasing discharge from cardiac mechanoreceptors with inhibitory vagal afferent [76].

### Inhibiting cardiac hypertrophy

Both prolonged exposure to systemic hypertension and lacking the inhibition of heart growth lead to cardiac hypertrophy [77, 78]. Moreover, ANP has direct effects on heart, and inhibits cardiac hypertrophy and fibrosis [79]. ANP induces cardiomyocyte apoptosis and inhibits fibroblast growth [3]. ANP suppresses fibroblast migration and proliferation by counteracting angiotensin-II, aldosterone and endothelin-1, as well as inflammatory reaction including pro-inflammatory factor and macrophage infiltration [80, 81]. ANP inhibits stress-activated protein kinase and extracellular-regulated kinase-2 induced by platelet-derived growth factor [82]. ANP exhibits antimitogenic and antineoplastic properties by reducing cell adhesion and inflammatory reaction through p38 mitogen-activated protein kinase [83]. ANP attenuates the growth of cardiomyocytes and fibroblasts by inhibiting norepinephrine-induced Ca^2+^ influx in a cGMP-mediated manner [84]. Meanwhile, ANP and BNP reduce systemic and pulmonary BP, and inhibit cardiac hypertrophy in HF [85]. ANP and CNP exert direct effects on vessels by reducing adhesion molecules of endotheliocytes (P-selectin and monocyte chemotactic protein-1) and inflammatory infiltration on atheromatous plaques [86].

### Regulating energy homeostasis

ANP stimulates exercise-induced lipolysis through cGMP and PKG in primates with an increased NPR-A/NPR-C ratio [87]. ANP affects the conversion from white to brown fat through mitochondrial uncoupling protein-1 and p38 mitogen-activated protein kinase [88]. BNP may also have hypoglycemic effect and regulate energy homeostasis [89]. ANP and BNP stimulate oxidative ability of skeletal muscle and lipolytic action in subcutaneous adipose [90]. ANP and BNP induce hormone-sensitive lipase of adipocytes in a cGMP-mediated manner [91].

### Function of BNP

In addition to the well documented natriuresis, diuresis and vasodilation, BNP also has direct effects on heart [14]. BNP may provide compensatory protection, such as inhibiting myocardial apoptosis and necrosis and reducing cardiac hypertrophy and fibrosis [92, 93]. BNP may also modulate immune and inflammatory reaction to cardiac injury. BNP depletes monocytes, B lymphocytes and natural killer cells in peripheral blood [94]. BNP regulates the chemotaxis of monocytes and production of inflammatory molecules by macrophages [95]. BNP may promote cardiac neutrophil infiltration and metalloprotease-9 expression after myocardial infarction (MI), and also have direct effects on matrix remodeling and wound healing [96].

### Affecting cardiac embryogenesis

BNP plays significant roles in cardiac embryogenesis. There are high BNP levels in embryonic heart during the midgestation, and peaks of BNP secretion correlate with cardiac development [97]. Plasma BNP levels in humans are high at birth, progressively declining thereafter, to stabilize at around ten years of age to the levels found in adults [98]. ANP and BNP may regulate cardiomyocyte differentiation and proliferation in the developing embryo [99]. Embryonic stem cells express high levels of BNP, which are crucial for their proliferation and differentiation [100]. BNP may also be involved in the process of angiogenesis following skeletal muscle ischemia [101]. BNP secretion by vascular satellite cells has been found to activate the regeneration of adjacent endothelium in a paracrine manner. Moreover, BNP has been addressed in cardiac regeneration by evaluating the relationships between cardiac precursor cells (CPCs) and BNP in neonatal and adult mice. Firstly, all forms of proBNP are more abundant in neonatal heart than in adult heart [102]. Secondly, CPCs express NPR-A and NPR-B, supporting that CPCs can respond to BNP [102]. NPR-A contributes to self-renewal and maintenance of CPC pluripotency, whereas NPR-B is involved in CPC proliferation [103]. ANP, BNP and CNP stimulate CPC proliferation and differentiation into new cardiomyocytes by NPR-B binding, cGMP increase and PKG activation [104]. Thirdly, exogenous BNP has increased proliferating CPCs and new cardiomyocytes, which are associated with improved cardiac function and remodeling after MI [102]. Finally, CPCs stain positive for BNP, suggesting that CPCs can also synthesize and secrete BNP in an autocrine manner to regulate their proliferation and differentiation into new cardiomyocytes. Thus, BNP and CPCs may be useful therapies for HF and MI [102].

### Function of CNP

All of ANP, BNP and CNP provide cardiorenal protection, although CNP has the most antifibrotic and least renal effects [13]. CNP is a vasodilator mainly secreted from endothelial cells in response to vascular injury. Patients with HF have minimally increased CNP levels, and HF severity is significantly relevant to CNP levels [105]. Although CNP dose not predominantly behave as a cardiac hormone, it has cardiovascular actions as well, such as re-endothelialization, hyperpolarization, antithrombosis and antifibrosis [106, 107]. CNP inhibits proliferation and migration of coronary artery smooth muscle cells mediated by oxidized low-density lipoprotein in a cGMP-dependent manner [108]. CNP inhibits platelet aggregation and thrombosis formation by suppressing plasminogen activator inhibitor-1, perhaps through NPR-C [109]. As an endothelium-derived hyperpolarizing factor, CNP may regulate several vasodilative factors, including prostacyclin and NO [110]. CNP inhibits cardiac hypertrophy and fibrosis in autocrine and paracrine manners within myocardium [111]. CNP has antifibrotic effect by regulating PKG-derived phosphorylation of Smad3 and transforming growth factor-β-derived nuclear translocation [112]. CNP may have compensatory actions in HF in a cAMP-dependent manner, which are incompletely understood. CNP-dependent NPR-B activity is about half of ANP-dependent NPR-A activity in normal ventricle. ANP-dependent NPR-A activity is unaltered or reduced, and CNP-dependent NPR-B activity is mildly or significantly elevated in failing ventricle [113, 114]. Failing ventricle has increased fibroblasts, and NPR-B is highly present in cardiac fibroblasts [115]. Additionally, CNP inhibits pulmonary hypertension and fibrosis in a similar manner in HF [116]. Fibroblast growth factor receptor 3 (FGFR3) is an important regulator of bone formation. Its gene gain-of-function mutations activate mitogen-activated protein kinase pathway and result in achondroplasia. CNP acts as a key regulator of longitudinal bone growth by downregulating mitogen-activated protein kinase pathway [117].

## Metabolism

### Mechanisms of metabolism

Main mechanisms are in the following: 1) NPR-C-derived and clathrin-mediated endocytosis, lysosomal ligand hydrolysis and ligand-free receptor recycling; and 2) NEP (neprilysin), a zinc-dependent exoenzyme with broad substrates [53]. ANP is degraded effectively in most organs, and more effectively in some than others. Although 30%–50% of ANP has been shown to be degraded in kidney, liver or lower limbs rather than lung, subsequent studies have reported that 19%-24% of ANP is degraded in lung (lung > liver > kidney) [118, 119]. Degradation of BNP and CNP have been discussed previously [120]. BNP binds to NPR-C 7% as tightly as ANP, and BNP has long half-life due to less degradation by NPR-C [121]. With a part of aa sequence similar to NPs, osteocrin (also known as musclin) is an important decoy ligand for NPR-C, and acts to increase plasma levels of NPs [122].

### NEP and IDE

NEP is a dominant enzyme for NP degradation. In heart, NEP is expressed on membrane of cardiomyocytes, fibroblasts, vascular smooth muscle and endothelial cells [123]. Although expressed in many epithelial tissues, NEP levels are particularly high at the luminal side of renal proximal tubules [124]. Initial attack of breaking ring and inactivating peptide occurs between cysteine and phenylalanine (Fig. 1) [125]. Cleavage sites in ring structure are crucial for degradation [48]. ANP and CNP have differences of zero or one aa between species, and are similarly degraded by NEP. BNP differs obviously between species, and is species-specifically degraded by NEP [126]. BNP is a worse substrate than ANP and CNP, and human NEP cleaves BNP at three sites [127]. Most BNP is degraded by NEP in rat renal membrane, but NEP dose not degrade all BNP in human renal membrane [128]. IDE, a zinc-dependent protease with broad substrates, degrades not only insulin, but also ANP [129, 130].

### Roles of NPR-C

Relative effects of NPR-C and NEP degradation on NP levels are still controversial and unclear [131]. Under normal condition, NPR-C-blocking peptides affect physiologic roles of ANP mildly more than or equally to NEP inhibitors, and ANP has maximal roles with both NPR-C-blocking peptides and NEP inhibitors [132]. Under pathologic condition, NEP inhibitors become important due to elevated NP levels and possible NPR-C saturation [133].

## Genetic regulation

### Genetic variants

Not only there are genetic variants in NPs and NPRs in humans, but also they have significant associations with cardiometabolic phenotypes [134]. NPPA gene has many variants in promoter, intronic, coding and 3’-UTR [134]. C-664G variant has been related to lower ANP levels, higher BP levels and more LV hypertrophy in Italian and Japanese populations [85]. Rs5063 variant has been related to lower BP levels in American and Chinese populations [135–137]. Rs5065 variant has been related to less hypertension and more MI [138–140]. However, lots of candidate genes have not been confirmed in big-sample population genetic studies and Genome Wide Association Studies. In a big-sample and genome-wide meta-analysis, rs5068 variant in 3’-UTR has been inversely regulated by micro-RNA (miR)-425 and related to higher ANP levels, lower BP levels and less LV hypertrophy [141]. Rs5068 variant has also been correlated with lower anthropometric indices, lower C-reactive protein, higher high density lipoprotein, as well as less susceptibility to HF [142–144]. In NPPB gene, rs198388 and 198389 variants have been related to lower BP levels, reduced LV remodeling, improved LV function and less diabetes mellitus [145, 146]. Variants in NPPC and NPR-B gene remain unclear [133]. Variants in NPR-C gene have been related to hypertension in Genome Wide Association Studies of Caucasian and Asian populations [147]. Variants in corin gene have been related to higher BP levels and more LV hypertrophy in African-American population [148].

### Genetic manipulation

In order to determine the function of NPs and medication of NPRs, genetic manipulation has been widely applied through the knockout of NPRs in animal experiments. For example, ANP-dependent natriuresis and diuresis are mediated exclusively by NPR-A in mice because these effects are completely lost after NPR-A knockout [62]. Mice lacking ANP or NPR-A have an enlarged heart, whereas mice over-expressing ANP have a smaller heart [79]. Mice over-expressing ANP are resistant to hypoxia-induced hypertension, whereas mice lacking ANP exhibit increased pulmonary hypertension in response to chronic hypoxia [73]. Moreover, designer NPs have been engineered through genetic alteration of native NPs. Compared with native NPs, designer NPs have improved efficacy and safety [7].

### Roles of micro-RNA

MiR-100 has been demonstrated to inhibit NPR-C expression in rat MI tissues and human LV cells, and increased miR-100 in HF may reflect a compensatory mechanism to prolong half-life of NPs [149]. MiR-425 inhibits ANP synthesis in human heart by interacting with 3’-UTR of NPPA gene, and antagonists of miR-425 may be a potential therapy for HF [144]. MiR-21 interacts with ANP in vascular smooth muscle cells through modulating downstream cGMP signaling [150]. MiR-30 is expressed in healthy heart, but suppressed in failing heart. MiR-30 inhibits GalNAc-transferase (GALNT) 1 and 2-mediated glycosylation of proBNP-108. MiR-30-GALNT pathway may be a novel therapeutic target for HF, and more researches are necessary in humans [32]. Although many miRs targeting NPRs have been suggested in silico analyses to modulate NP signaling pathways in HF, it is necessary to confirm their actual interactions in vivo experiments [151].

### Epigenetic remodeling

Maladaptive remodeling of LV in HF is correlated with fetal-gene reactivation and epigenetic remodeling in promoters of NPPA and NPPB genes. Although there is nuclear export of histone deacetylase 4 (HDAC4), gene activation of NPPA and NPPB dose not require increased histone acetylation in promoters. In contrast, methylation of histone 3 lysine 9 (H3K9) and binding of heterochromatin protein 1 (HP1) in promoters of these genes are reduced by HDAC4, perhaps by forming a transcriptional repressor complex with histone methyltransferase (suppressor of variegation 3-9 homolog 1, SUV39H1). This complex is disrupted by Ca^2+^/calmodulin-dependent kinase II (CaMKII)δB-induced phosphorylation of HDAC4. Histone demethylase [Jumonji C (JmjC) domain-containing demethylase] may be upregulated to maintain H3K9 demethylation in HF [152].

## Diagnostic values

### Practical application

NPs reflect cardiac stress and function, increase drastically in patients with HF, and have powerful diagnostic value for various forms of HF [153]. Plasma NP levels are also used to evaluate HF severity [154, 155]. Plasma ANP levels differ according to atrial pressure, whereas plasma BNP levels reflect ventricular overload. BNP (half-life: 22 minutes) has been shown to have greater stability than ANP (half-life: 2 minutes) [156]. Both BNP and NT-proBNP are removed by kidney, and BNP is also degraded by NEP and NPR-C. BNP has shorter half-life than NT-proBNP (half-life: 70 minutes). Thus, BNP and NT-proBNP are preferred to other NPs as gold standard for HF diagnosis, and established as rule-out tests of HF based on clinical guidelines [157, 158]. LV hypertrophy and dysfunction lead to higher levels of ANP and BNP. Thus, elevated ANP and BNP levels can be used to identify LV hypertrophy and dysfunction in general population and hospitalized patients [159]. Rapid assay for NPs can not only discriminate the origin of acute dyspnea (acute HF versus bronchial asthma), but also manage the patients with chronic HF [160–162]. Plasma NT-proBNP level 300 pg/ml is appropriate for ruling out acute HF. Age-dependent cutoff levels of plasma NT-proBNP are appropriate for ruling in acute HF: 450 pg/ml in patients < 50 years of age, 900 pg/ml in patients ≥ 50 years of age, and 1800 pg/ml in patients > 75 years of age [163]. Plasma BNP level 100 pg/ml and 400 pg/ml are appropriate for ruling out and ruling in acute HF, respectively. Cutoff levels of plasma NT-proBNP and BNP are 125 pg/ml and 35 pg/ml to chronic HF, respectively [164].

### Confounding factors

Although applying BNP and NT-proBNP as diagnostic biomarkers of HF has brought significant improvement in treating HF, several confounding factors, such as aging, obesity, anemia, sepsis, hypertension, MI, cardiac hypertrophy, pulmonary hypertension, atrial fibrillation, diabetes mellitus, renal failure, liver cirrhosis, severe burn and cancer chemotherapy, limit their accuracy [165]. Plasma NP levels have been inversely related to body mass index in epidemiological investigations [166, 167]. Plasma NPs have lower levels in patients with diabetes mellitus, insulin resistance or metabolic syndrome, perhaps contributing to HF risk [168, 169]. Plasma BNP has higher levels in patients with hypertension or LV hypertrophy than in those without them [170, 171]. Plasma BNP has higher levels in patients with LV concentric hypertrophy than in those with LV eccentric hypertrophy or in those with normal LV structure and hypertension [172]. BP-lowering therapy reduces BNP levels and LV mass [173, 174].

### Coronary artery disease

Plasma NPs have higher levels in patients with acute coronary syndrome or exercise-induced myocardial ischemia but without ventricular dilation [175]. Plasma ANP levels rise until admission and then decline in patients with acute MI. Plasma BNP levels rise until 12-24 hours after acute MI, then decline and peak once more after 5-7 days [176]. Height of the second peak is an useful indicator of LV remodeling [177]. Although there is a gradual decrease, plasma BNP levels are still increased in chronic phase, showing LV damage and remodeling [178]. However, plasma BNP has almost normal levels if early coronary reperfusion successfully prevents LV remodeling.

### Chronic kidney disease

Renal function has systematical effects on NP system. In patients with chronic kidney disease (CKD), plasma NP levels are elevated as compensatory protection of renal function. CKD is always related to cardiovascular abnormalities. However, due to unclear mechanisms, there are elevated BNP levels in patients with CKD but without cardiovascular abnormalities [179]. Plasma NP levels may be regulated both by synthetic/secretory rate from heart and by extraction rate from blood. Key mechanism may not be an increase in extraction rate from kidney. Other mechanisms include the decreases in functional renal mass, second messenger synthesis and clearance receptor degradation in kidney [180, 181]. Elevated NP levels in CKD are correlated with a counter-regulatory response directed from heart to kidney, suggesting NPs as potential biomarkers of LV remodeling in patients with CKD [181]. Plasma NP levels in CKD reflect the stress on cardiac wall caused by LV hypertrophy or dysfunction [182].

### End-stage renal disease

Plasma BNP and NT-proBNP have higher levels in patients with end-stage renal disease. There is a decrease of about 20–40% in plasma BNP levels after hemodialysis (HD). Peritoneal dialysis (PD) may not alter plasma BNP levels [183]. HD promotes fluid clearance and alleviates volume overload, leading to reduced wall stress and NP release [184]. Plasma BNP has lower levels in patients with PD than in those with HD, supporting that PD may lead to slower ultrafiltration rate, higher urine output, better hemodynamic condition, less cardiac load and lower BP levels than HD [185]. However, it remains inconclusive and needs more researches [186]. Meanwhile, due to reduced ultrafiltration rate, continuous volume overload and more LV hypertrophy, patients with automated PD (APD) may have higher BNP levels than those with continuous ambulatory PD (CAPD) [187]. Moreover, plasma BNP levels have a significant potential to identify LV hypertrophy or dysfunction in patients with different dialysis and renal transplant [188–190].

### Practical application in CKD

Renal function limits current use of NPs in patients with CKD [191]. Plasma NP levels in patients with CKD are related to CKD severity, and cutoff levels are increased as CKD stages advance. Plasma BNP levels rise to almost 200 pg/ml in patients with CKD but without HF. Compared with plasma BNP levels, plasma NT-proBNP levels may be more strongly correlated with GFR and affected by age-related decrease in GFR, suggesting careful use of NT-proBNP in elderly with CKD [192]. In patients with CKD, plasma NT-proBNP levels > 1200 pg/ml suggest chronic HF in patients < 50 years of age and > 4502 pg/ml in patients between 50 and 75 years old [180]. It remains unclear if elevated NP levels in CKD effectively reflect the activation of NP system and effects on target organ. Elevated NP levels may have reduced ability to activate NP system and affect target organ in CKD. NP resistance in CKD may be caused by downregulated NPR-A expression in renal medulla and upregulated NPR-C expression in renal cortex [193]. NP resistance in CKD results in the invalidity of NP infusion in protecting renal function and treating cardiorenal syndrome in patients with HF [194].

### Point-of-care systems

Previous assays for BNP are invasive and time-consuming with the discomfort caused by venipuncture. Some ideal point-of-care (POC) systems have been developed to allow rapid and repeated assays for BNP from capillary blood, like measuring blood sugar from fingertip [195]. A system at bedside would not only be useful for repeated assays at home, but also achieve routine monitoring in a remote way. Additionally, POC systems would also be used in the hospitals for BNP-guided therapy of HF and rapid triage of dyspnea. Two POC systems for BNP use either venipuncture ethylenediaminetetraacetic acid (EDTA) plasma (Alere Triage) or EDTA whole blood (Abbott i-STAT) [196–199]. In contrast, Alere Heart Check BNP Test is the first POC system easily used as a rapid assay for BNP from untreated fingertip capillary whole blood. Previous studies have demonstrated the safety and feasibility of Alere Heart Check BNP Test at home [200–202].

### Current bioactivity assays

Current immunoassays can not reflect the bioactivity of NPs [203]. Rabbit aortic strip test (RAST) measures inhibitory activity of recombinant form of human BNP (rhBNP) on the tension of isolated aortic strip to determine its bioactivity [204]. However, it is variable, rough, laborious and time-consuming with the isolation of fresh issues from sacrificed rabbits. As the well-characterized pathways activated by rhBNP, cGMP has been quantified as alternative assays in human umbilical vein endotheliocyte (HUVEC) or rat pheochromocytoma cell-12 (PC-12) by radioimmunoassays [205]. However, HUVEC is primary cell and PC-12 tends to differentiate. Both HUVEC and PC-12 are not stable in culture. These intracellular cGMP measurements have limited accuracy, precision and reproducibility. They are tedious with the preparation of cell lysate and depend on standard curve by radioimmunoassays [206]. One stable cell-based assay is based on the NPR-A in HEK 293 cell. This single high-responsiveness clone is simplified by detecting the cGMP in culture supernatant with non-radioactive material in a high-throughput manner [207]. Although this assay has a significant potential to monitor endogenous activities of NPs, it needs more researches involving different NP forms and clinical stages [203].

## Therapeutic values

### Mechanisms of therapy

Mortality rate remains high in patients with HF even with the best current therapies. This mandates a continuing search for new therapies. NPs counteract RAAS by inhibiting renin secretion through second messenger cGMP, and NP augment on top of RAAS blockade may have synergistic effects on HF [208]. NPR-A suppression activates the RAAS and impairs the kidney [209]. Although HF develops with progressive activation of NPs, this response is apparently insufficient to counteract vascular constriction and sodium retention of RAAS and SNS [7]. Synthetic ANP has an attenuated renal response (natriuresis and diuresis) in patients with HF, suggesting NPR dysregulation in these patients with RAAS activation [62]. However, synthetic ANP has other significant roles, such as hemodynamic improvement and RAAS inhibition, in patients with HF [210]. Elevated NP levels maintain sodium balance in early stage of HF, and NPR-A suppression in HF causes sodium retention [211, 212]. NPs suppress angiotensin-II-induced vasoconstriction, angiotensin-II-stimulated proximal tubule sodium reabsorption, angiotensin-II-enhanced aldosterone secretion and endothelin secretion [213]. Moreover, most of plasma BNP measured with current immunoassays is less active in patients with HF. Thus, HF induces an attenuated response to elevated BNP levels, and represents a deficiency of active BNP caused by abnormal processing of NPs [214, 215].

### Increasing NP bioactivity

Novel therapies are under development based on an augment in cardioprotective effects of NPs to re-balance neuroendocrine dysregulation in HF [216]. Current NP-augmenting strategies include the synthesis of NPs or agonists to increase NP bioactivity and inhibition of NEP to reduce NP breakdown [217, 218]. Nesiritide, a rhBNP, has been shown to induce hemodynamic and clinical improvements in Vasodilatation in the Management of Acute CHF (VMAC) and other trials [219]. Nesiritide is successfully approved by Food and Drug Administration (FDA) and routinely used for both acute and chronic HF. However, nesiritide has been questioned in two subsequent meta-analyses to worsen renal function and increase mortality rate [220]. Other studies, such as Acutely Decompensated Heart Failure Registry (ADHERE) trial, have not confirmed these unfavorable effects of nesiritide [221, 222]. Acute Study of Clinical Effectiveness of Nesiritide in Decompensated Heart Failure (ASCEND-HF) trial has reported that nesiritide has no significant relationship with mortality rate, nor is it related to worsening renal function [223]. Neutral conclusions may be correlated with nesiritide dose. Large dose of nesiritide is strongly vasodilative, causes severe hypotension and neutralizes beneficial roles. Recent studies have reported that small dose of nesiritide, particularly when administered through subcutaneous route, induces hemodynamic and clinical improvements, without increasing nephrotoxicity and mortality, thus reopening the debates about its usefulness in patients with HF [224]. Long-term antiapoptotic, antiremodeling and antihypertrophic actions of nesiritide are beneficial if NPR-A could be activated chronically [225]. Nesiritide administered twice daily for eight weeks through subcutaneous route has improved clinical symptoms and reduced LV mass in patients with HF [226]. Additionally, nesiritide has been suggested to protect LV function in patients with MI [227]. Carperitide, recombinant form of human ANP, has alleviated clinical symptoms and been recommended in Japan for acute decompensated HF [125]. But short half-life limits clinical application of carperitide. Moreover, oral forms of ANP and BNP are too unstable to be routinely used in HF. CNP is unsuitable for treating HF due to relatively short half-life and no renal-enhancing action. In Safety and Efficacy of an Intravenous Placebo-controlled Randomised Infusion of Ularitide (SIRIUS I and II), synthetic urodilatin (ularitide) has induced hemodynamic and clinical improvements, without worsening renal function and obvious BP change, in patients with acute decompensated HF [228, 229]. Another Phase III Trial of Ularitide Efficacy and Safety in Acute Heart Failure (TRUE-AHF) has shown that ularitide reduces systolic BP and cardiac stress as indicated by plasma NT-proBNP levels, but has no effect on clinical composite end point, cardiovascular mortality and myocardial injury as indicated by cardiac troponin T levels [230].

### Designer NPs

Severe hypotension and short half-life make recombinant agents, including nesiritide, carperitide and ularitide, not very suitable for clinical use. Designer NPs are developed by altering genetic forms or aa structures of native NPs. These hybrid peptides have normal binding to NPR-A and increased resistance to degradation [7]. DNP is firstly discovered in snake venom, and much about DNP remains unclear in humans. Cenderitide-NP (CD-NP) is not easy to be degraded as a 37-aa hybrid NP designed by fusing native CNP-22 with 15-aa C-terminal of DNP. This first-generation designer NP retains vasodilative, antifibrotic and antihypertrophic roles of CNP, and natriuretic and diuretic roles of DNP [231]. Both NPR-A and NPR-B can be effectively activated by CD-NP to increase more GFR and cause less hypotension than nesiritide, with reduced atrial pressure and improved cardiac-unloading effect [232]. FDA has provided a fast-track designation for CD-NP in Phase II trials [233]. CU-NP has been designed by fusing 17-aa ring structure of native CNP with C- and N-terminal of urodilatin [234]. As an experimental agent in early stage, CU-NP exerts cardiac-unloading, renal-enhancing and RAAS-suppressing effects through activating cGMP. CU-NP has direct antihypertrophic effect through inhibiting sodium-hydrogen exchanger 1 (NHE-1)/calcineurin pathway [235]. Mutant ANP (M-ANP) has been designed as a 40-aa peptide by fusing native ANP with 12-aa extension to C-terminal [236]. M-ANP has exerted beneficial cardiac and renal effects, such as boosting natriuresis and diuresis, regulating BP and GFR, inhibiting RAAS and SNS, and promoting antifibrosis and antiproliferation in experiments [237]. Novel NPs are currently under clinical development programs for further trials [238]. An alternative RNA spliced transcript for BNP (AS-BNP) has a unique 34-aa C-terminal, with remaining structure of native BNP [205]. ANX-042 has been designed as a 42-aa peptide by fusing 16 aa from C-terminal of AS-BNP and 26 aa from native BNP. ANX-042 can activate cGMP to boost natriuresis and diuresis and suppress renin and angiotensin-II, but not activate cGMP to relax blood vessels. As a designer NP in a first-in-human trial, FDA has suggested ANX-042 as an investigated new drug for HF with renal protection and less hypotension [239]. CNP analog (BMN111) is one of the most promising therapy for achondroplasia, and obviously improves skeletal parameters in animal experiments [240].

### Reducing NP degradation

Although NP breakdown can be blocked by affecting NPR-C and inhibiting IDE, the more commonly used approach to reduce NP degradation is NEP inhibition. However, there are plentiful substrates of NEP, such as angiotensin-I, angiotensin-II, bradykinin, substance P, adrenomedullin, endothelin-1, opioid peptide, insulin β-chain, glucagon, oxytocin, chemotactic peptide, neurotensin, enkephalins, gastrin and amyloid-β peptide. NEP inhibition has potential to increase levels of these substrates, leading to conflicting effects on kidney and vessels [7]. Moreover, NEP hydrolyzes angiotensin-I to angiotensin-(1-7), which counteracts angiotensin-II. As the first pure NEP inhibitor, candoxatril is stable when administered orally. However, due to its effects on other systems, candoxatril has no benefit for patients with hypertension or HF [241]. Candoxatril is characterized by both NP augment (elevated NP levels and natriuresis) and RAAS activation (elevated angiotensin-II levels and vasoconstriction), leading to unaltered vascular resistance and unavailable antihypertensive role.

### Dual ACE/NEP inhibitors

Pure NEP inhibitors have disappointing clinical effects, which may be improved by combining RAAS blockade. Due to no improvement in clinical symptoms and an increase in aplastic anemia, it is unpractical to choose an addition of NEP inhibitors (ecadotril) to standard therapies including angiotensin-converting enzyme (ACE) inhibitors [242]. Dual ACE/NEP inhibitor (vasopeptidase inhibitor, sampatrilat) has shown beneficial effects, but then been dropped due to short half-life [243]. Omapatrilat (BMS-186716) has almost affinity and inhibition for NEP and ACE. In experimental HF and hypertension models, omapatrilat has not only improved clinical symptoms and survival, but also relieved cardiac dysfunction and hypertension [244]. Moreover, omapatrilat has improved cardiac function and remodeling, and decreased cardiac hypertrophy and fibrosis in mice with MI [245]. Omapatrilat has been evaluated in patients with HF or hypertension in Omapatrilat Cardiovascular Treatment Versus Enalapril (OCTAVE), Inhibition of Metalloprotease by Omapatarilat in a Randomized Exercise and Symptoms Study of Heart Failure (IMPRESS) and Omapatrilat Versus Enalapril Randomized Trial of Utility in Reducing Events (OVERTURE) trials [246–248]. Omapatrilat has more obviously lowered vascular resistance and BP levels than candoxatril. As bradykinin is degraded by both NEP and ACE, simultaneous inhibition of them by omapatrilat increases bradykinin levels that favor the development of angioedema. Compared with enalapril, omapatrilat has increased angioedema and hypotension, and shown no superior benefit in patients with HF or hypertension, precluding its clinical use and final approval of FDA [249].

### Triple ACE/ECE/NEP inhibitors

Endothelin-1 is a multifunctional vasoconstrictor and contributes to HF progression [250]. Most endothelin-1 receptor antagonists have no prognostic improvement in patients with acute and chronic HF. In experimental HF model, endothelin-converting enzyme (ECE) inhibition has suppressed endothelin-1 synthesis, improved cardiorenal function and reduced the neurohormones, such as renin, angiotensin-II and aldosterone [251]. NPs may be degraded by ECE. Thus, ECE inhibition may simultaneously augment the NPs and suppress the endothelin [252]. ECE inhibition has induced hemodynamic improvement in patients with HF [253]. However, there is no long-term study about ECE inhibition. Dual ECE/NEP inhibitor SLV-306 (daglutril) has not only lowered LV pressure in patients with HF, but also improved cardiac function and remodeling in rats with LV hypertrophy [254]. Moreover, daglutril has inhibited BP elevation and increased NP levels in healthy humans [255]. Another dual ECE/NEP inhibitor (SLV-338) has improved cardiac fibrosis in experiment [256]. Triple ACE/ECE/NEP inhibitors may inhibit the synthesis of angiotensin-II and endothelin-1, and enhance the effects of NPs and bradykinin. In experimental HF model, triple ACE/ECE/NEP inhibition has been superior to ACE inhibition and dual ECE/NEP inhibition in improving LV structure and function [257]. However, the development of triple ACE/ECE/NEP inhibitors may be obstructed by negative conclusions about endothelin-1 receptor antagonists from large HF trials and practical concerns about the safety with ACE/NEP inhibitors. It needs to be emphasized that endothelin-1 receptor antagonism and ECE inhibition should be distinguished in further human trials.

### Development of LCZ696

Angiotensin receptor blocker NEP inhibitor (ARNI, LCZ696) is a major advance in the therapies for HF in the last 15 years. Molecular moieties of NEP inhibitor prodrug sacubitril (AHU377), and valsartan, an angiotensin receptor blocker (ARB), are present in this single molecule in 1:1 molar ratio (sacubitril/valsartan). Sacubitril (AHU377) becomes active NEP inhibitor LBQ657 after cleaving ethyl ester. ARNI preserves ACE mechanism for bradykinin degradation [258]. ARNI augments beneficial effects of NPs and inhibits harmful effects of angiotensin-II. As the first-in-class ARNI, LCZ696 has improved cardiac dysfunction, fibrosis, remodeling and hypertrophy in an animal model [245]. Compared with valsartan alone, LCZ696 has more effectively lowered BP levels, without increased angioedema, in patients with hypertension [259]. In patients with HF with reduced ejection fraction (HFrEF), LCZ696 has more effectively reduced all-cause, cardiovascular and sudden death, prevented HF progression and hospitalization, and improved life quality and renal function than enalapril in Prospective comparison of ARNI with ACE inhibitor to Determine Impact on Global Mortality and morbidity in Heart Failure (PARADIGM-HF) trial [260–263]. LCZ696 has recently been approved by FDA for treating HFrEF. But translating the results of this trial into guideline recommendation has raised some concerns [264]. Firstly, this study has been discontinued ahead of schedule due to overwhelming benefit of LCZ696, and there is a doubt about efficacy and safety of LCZ696 used for longer time [265]. Secondly, LCZ696 was administered twice daily at a dose of 200 mg (160 mg of valsartan), and enalapril was administered twice daily at a dose of 10 mg. Both two doses are target doses in most HF guidelines but higher than many patients with HFrEF may tolerate. Thirdly, more symptomatic postural hypotension in LCZ-696 group limited its clinical use, particularly in patients with borderline BP before therapy. It is necessary to observe this agent according to baseline BP in further studies. Fourthly, bradykinin levels have previously been shown to increase with ARB, and there were more patients with angioedema in LCZ-696 group [266]. Fifthly, amyloid-β peptide is a key peptide in Alzheimer disease, and NEP may block its breakdown to induce Alzheimer disease [267]. However, Alzheimer disease and cancer were not increased using LCZ696, and cognitive decline related to vascular diseases may be reduced by LCZ696. Finally, drug denials have already been increased, and applying LCZ696 would be further complicated by cost. Compared with valsartan, LCZ696 has not only reduced NT-proBNP levels and caused GFR elevation, but also improved overall clinical status and decreased left atrial pressure in Prospective comparison of ARNI with ARB on Management Of heart failUre with preserved ejectioN fracTion (PARAMOUNT) trial [268]. Whether it would benefit patients with HF with preserved ejection fraction (HFpEF) is currently being tested in ongoing Efficacy and Safety of LCZ696 Compared to Valsartan on Morbidity and Mortality in Heart Failure Patients With Preserved Ejection Fraction (PARAGON-HF, NCT01920711) trial. ARNI class has sparked considerable excitement in other cardiovascular diseases including hypertension [269]. Prospective comparison of Angiotensin Receptor neprilysin inhibitor with Angiotensin receptor blocker MEasuring arterial sTiffness in the eldERly (PARAMETER) trail is also underway to compare the relationships of LCZ696 and olmesartan with central BP in patients with resistant hypertension [270].

### Mechanisms of LCZ696

It remains unclear about the mechanisms responsible for the superiority of LCZ696 over ACE inhibitor. Considering elevated levels of plasma BNP and urinary cGMP, systemic vasodilation and renal natriuresis could be important mechanisms. But it remains uncertain whether its superiority is direct effect on heart or indirect effect secondary to beneficial effects of this agent on vessels (lower BP levels) and kidney (less renal injury) [271]. Lower NT-proANP and troponin levels support that LCZ696 directly reduces myocardial stretch or ischemia. LCZ696 reduces proteinuria, focal segmental glomerulosclerosis and retinopathy, and plays beneficial effects on microvascular and renal complications. Without the evidence in PARADIGM-HF, further studies are required to fully address this issue. Meanwhile, positive effects of BNP on cardiac regeneration may also play an important role, and should be fully addressed in ongoing experimental and clinical studies. Exogenous BNP or NEP inhibition may induce endogenous cardiac regeneration, and achieve the therapies for HF and MI [4]. In experimental hypertension models, either alone or combined with MI or diabetes mellitus, ARNI has improved cardiac hypertrophy and fibrosis in a BP-independent manner. Since sacubitril is largely cleared in kidney, drug accumulation may occur in patients with impaired renal function, and hypotension is a potential adverse effect in patients with CKD.

### BNP-guided therapy

Serial assays for BNP over time are clinically applied to the management of HF. BNP-guided therapy can assess the effectiveness and adjust the doses of drugs for HF, and improve the survival in patients with HFrEF or HFpEF [272]. United Kingdom-based economic model of BNP-guided therapy has been developed in patient with chronic HF [273]. BNP-guided therapy is cost-effective in younger patients (< 75 years) with HFrEF. It is potentially cost-effective in younger patients (< 75 years) with HFpEF and older patients (≥ 75 years) with HFrEF, but more evidence is required, particularly with respect to the frequency, duration and target for BNP monitoring [274]. Ongoing Guiding Evidence Based Therapy Using Biomarker Intensified Treatment in Heart Failure (GUIDE-IT) trial would be very important in providing better evidence in patients with HFrEF [275]. Additionally, NEP inhibitors may increase BNP levels and lower NT-proBNP levels, and require different monitoring strategies in BNP-guided therapy [261].

## Prognostic values

Plasma NP levels have prognostic values in patients with cardiovascular diseases. Previous studies on NP infusion, experimental animals and population genetics have demonstrated inverse correlations of plasma NP levels with different cardiovascular diseases. But epidemiological and clinical investigations of NPs as prognostic biomarkers have yielded positive correlations of plasma NP levels with poor prognosis [276]. As counter-regulatory hormones secreted after cardiac stretch, NPs have this paradox should be no surprise. In epidemiological investigations, elevated NP levels even in regular limit have been commonly observed in patients with subclinical cardiovascular diseases. In Framingham Offspring Study and Copenhagen, elevated NP levels have been significantly related to major adverse cardiovascular events and mortality rate in population without obvious cardiovascular diseases [277]. In patients with stable coronary artery disease, acute coronary syndrome or HF, elevated NP levels have also been significantly associated with cardiovascular events and mortality rate [154, 278, 279]. If plasma BNP or NT-proBNP levels do not fall off after the therapies for HF, patients with HF have more hospital admission and higher mortality rate. In patients with HF, NT-proBNP has higher levels, better accuracy, longer half-life and lower variation than BNP, and may be a better biomarker of HF progression and mortality rate [280]. Moreover, NPs are reliable predictors of all-cause and cardiovascular death independently of other clinical and biochemical risk factors, and have a potential to guide the therapy and predict the prognosis in patients with CKD [281–283].

## Conclusion

NPs play central roles in the regulation of HF. Both BNP and NT-proBNP are useful biomarkers to not only make the diagnosis and assess the severity of HF, but also guide the therapy and predict the prognosis in patients with HF. Current NP-augmenting strategies include the synthesis of NPs or agonists to increase NP bioactivity and inhibition of NEP to reduce NP breakdown. Nesiritide has been established as an available therapy, and ARNI has obtained extremely encouraging results with decreased morbidity and mortality. Novel pharmacological approaches based on NPs may promote a therapeutic shift from suppressing the RAAS and SNS to re-balancing neuroendocrine dysregulation in patients with HF.

## Abbreviations

Aa: Amino acid
ANP: Atrial natriuretic peptide
APD: Automated peritoneal dialysis
ARNI: Angiotensin receptor blocker NEP inhibitor
BNP: B-type natriuretic peptide
BP: Blood pressure
CAPD: Continuous ambulatory peritoneal dialysis
CD-NP: Cenderitide-NP
cGMP: Cyclic guanosine monophosphate
CKD: Chronic kidney disease
CPCs: Cardiac precursor cells
CNP: C-type natriuretic peptide
C-terminal: Carboxy-terminal
DPP-4: Dipeptidyl peptidase-4
GC: Guanylate cyclase
GFR: Glomerular filtration rate
GTP: Guanosine triphosphate
HD: Hemodialysis
HF: Heart failure
IDE: Insulin degrading enzyme
LV: Left ventricle
miR: micro-RNA
NEP: Neutral endopeptidase
NO: Nitric oxide
NP: Natriuretic peptide
NPP: Natriuretic peptide precursor
NPR: Natriuretic peptide receptor
N-terminal: Amino-terminal
NT-proANP: N-terminal proANP
NT-proBNP: N-terminal proBNP
PD: Peritoneal dialysis
PDE: Phosphodiesterase
PKG: Protein kinase
POC: Point-of-care
RAAS: Renin-angiotensin-aldosterone system
rhBNP: Recombinant form of human BNP
SNS: Sympathetic nervous system
5’-FR: 5’-flanking region
5’-UTR: 5’-untranslated region
